# Prenatal Sociodemographic Factors Predicting Maltreatment of Children up to 3 Years Old: A Prospective Cohort Study Using Administrative Data in Japan

**DOI:** 10.3390/ijerph18052505

**Published:** 2021-03-03

**Authors:** Aya Isumi, Kunihiko Takahashi, Takeo Fujiwara

**Affiliations:** 1 Department of Global Health Promotion, Tokyo Medical and Dental University (TMDU), Tokyo 113-8519, Japan; fujiwara.hlth@tmd.ac.jp; 2 Japan Society for the Promotion of Science, Tokyo 102-0083, Japan; 3 Medical and Dental Data Science Center, Tokyo Medical and Dental University (TMDU), Tokyo 113-8519, Japan; kunihikot.dsc@tmd.ac.jp

**Keywords:** child maltreatment, pregnancy, risk assessment, prevention

## Abstract

Identifying risk factors from pregnancy is essential for preventing child maltreatment. However, few studies have explored prenatal risk factors assessed at pregnancy registration. This study aimed to identify prenatal risk factors for child maltreatment during the first three years of life using population-level survey data from pregnancy notification forms. This prospective cohort study targeted all mothers and their infants enrolled for a 3- to 4-month-old health check between October 2013 and February 2014 in five municipalities in Aichi Prefecture, Japan, and followed them until the child turned 3 years old. Administrative records of registration with Regional Councils for Children Requiring Care (RCCRC), which is suggestive of child maltreatment cases, were linked with survey data from pregnancy notification forms registered at municipalities (*n* = 893). Exact logistic regression was used for analysis. A total of 11 children (1.2%) were registered with RCCRC by 3 years of age. Unmarried marital status, history of artificial abortion, and smoking during pregnancy were significantly associated with child maltreatment. Prenatal risk scores calculated as the sum of these prenatal risk factors, ranging from 0 to 7, showed high predictive power (area under receiver operating characteristic curve 0.805; 95% confidence interval (CI), 0.660–0.950) at a cut-off score of 2 (sensitivity = 72.7%, specificity = 83.2%). These findings suggest that variables from pregnancy notification forms may be predictors of the risk for child maltreatment by the age of three.

## 1. Introduction

Child maltreatment is one of the most important social issues that require urgent attention in Japan, considering the steady increase in the number of child maltreatment cases handled at Child Guidance Centers [1] and the long-term detrimental impact on the child’s physical and mental health [2,3,4,5,6]. In Japan, when a child is judged as requiring protection or social support due to child maltreatment, the child is registered with Regional Councils for Children Requiring Care (RCCRC). Once registered, RCCRC hold conferences with stakeholders, such as Child Guidance Centers, schools, medical institutions, or police; collect information about the child and his/her family; assess the degree of emergence and seriousness of the child’s situation; develop a support plan; and monitor the case. A total of 61,117 children were newly registered with RCCRC due to child maltreatment in a fiscal year (FY) 2016 [7], which accounts for approximately 0.3% of children aged 0 to 18 years old in Japan [8].

Children in the first three years of life are at the highest risk for maltreatment [9]. In particular, children under 1 year old accounted for half of the deaths from child maltreatment (except filicide–suicide) in 2007–2016 in Japan [10]. Given the vulnerability of young children, it is critical to identify risk factors for primary prevention of child maltreatment and to provide support to families at risk as early as possible, ideally during pregnancy. Previous population-based studies in the United States have explored perinatal sociodemographic risk factors for the prediction of child maltreatment during the first three to five years of life by linking child welfare data (i.e., child protective service records) with birth record data [11,12] or data from prenatal risk assessment [13,14,15]. The risk factors identified during pregnancy include young maternal age, low maternal education, unmarried marital status, smoking, drinking, and substance use during pregnancy, having two or more older children, abortion history, short interpregnancy interval (i.e., less than 18 months), Medicaid beneficiaries, intimate partner violence (IPV), and inadequate prenatal care [11,12,13,14,15]. However, to our knowledge, no population-based studies have used administrative data from pregnancy registration systems and objective data on child maltreatment in Japan to explore prenatal sociodemographic risk factors for child maltreatment.

In Japan, all pregnant women are encouraged to register pregnancy notification forms at municipalities as soon as their pregnancy is confirmed to ensure that they receive Maternal and Child Health Handbooks and publicly funded tickets for health checks. Pregnancy notification forms often include questionnaires that assess sociodemographic information about the pregnant women and their families. Many municipalities in Japan utilize such questionnaires to identify pregnant women at risk in the early stage of pregnancy, and public health nurses contact all of them and provide support via telephone calls, interviews, and home visitation throughout their pregnancy if they are willing to receive services. They listen to pregnant women’s concerns, provide information and advice for healthy pregnancy, and connect them to services they may need. Intensity (i.e., frequency and duration) and content of such services depend on the assessment results and resources of the municipality (i.e., number of public health nurses). However, assessment items in pregnancy notification forms vary from municipality to municipality and are not digitized in all municipalities. Even if digitized, it is not easy to link with future data on children due to data management systems. Therefore, these assessment items in pregnancy notification have not been evaluated for their predictability of actual future child maltreatment. To promote effective prevention efforts starting from pregnancy, it is important to conduct evidence-based risk assessment of pregnant women and their families.

In Aichi Prefecture, located in the middle of Japan, with the fourth largest population [16] and third highest prefectural income among Japanese prefectures in 2013 [17], the same questions are used on pregnancy notification forms across the prefecture and most of them are digitized. In addition, some municipalities in Aichi Prefecture allowed us to link their data with those of the RCCRC. The universal pregnancy registration system in Japan covers a large proportion of the population, with more than 99% of pregnant women estimated to register pregnancy notification forms at municipalities before delivery in Japan [18]. This is in comparison to the approximately 70% of pregnant women who were screened between 1999 and 2005 through the Healthy Start Prenatal Risk Screen, a universal pregnancy screening program in Florida, United States [19]. Thus, this study aimed to examine prenatal sociodemographic risk factors for child maltreatment from birth to age three, using survey data from pregnancy notification forms linked with data from the RCCRC.

## 2. Materials and Methods

### 2.1. Participants

This study targeted mothers of infants who were enrolled for a 3- to 4-month health check between October 2013 and February 2014 in five municipalities in Aichi Prefecture and followed them until a 3-year health check (*n* = 994). The total population of the five municipalities, Toyokawa City, Handa City, Gamagori City, Tahara City, and Oguchi Town, was 465,809, with 3900 births in FY2014. Of the target population, data from pregnancy notification forms registered at the five municipalities were available and retrospectively collected from 893 mothers. Mothers whose pregnancy notification forms were unavailable (*n* = 101) were those who moved from other municipalities after submitting their pregnancy notification forms to the other municipalities. The data from pregnancy notification forms were prospectively linked with administrative records of registration with RCCRC until the child turned three years old, using household ID numbers, and analyzed. Only in these five municipalities, data from pregnancy notification forms were able to link with administrative records of registration with RCCRC, and therefore this sample served as an examination of the feasibility of linking of data.

### 2.2. Measurements

#### 2.2.1. Child Maltreatment

In this study, registration with RCCRC was regarded as a child maltreatment case, given that children are registered with RCCRC when they are judged as requiring protection or care due to child maltreatment in Japan. Whether a child was ever registered with RCCRC up to the age of three years was used as an outcome variable. Children who were registered with RCCRC in this study included both children in need of protection (*yohogo-jido* in Japanese) and those requiring social support (*yoshien-jido* in Japanese).

#### 2.2.2. Prenatal Risk Factors

Pregnancy notification forms included information on the following: marital status (“married” or “unmarried”), maternal age at submission of the pregnancy notification, gestational weeks at registration, pregnancy progress (“good” or “not good”), birth order (“first” or “subsequent”), history of artificial (or induced) abortion (“yes” or “no”), history of infertility treatment (“yes” or “no”), feelings when notified of the pregnancy (“happy”, “unexpected but happy”, “unexpected and puzzled”, “did not know what to do”, “no feeling”, or “other”), returning to parents’ home for delivery (“yes” or “no”), having someone who can help when in need (“yes” or “no”), having worries or anxiety (“yes” or “no”), smoking before/during pregnancy (“yes,” “stopped after pregnancy was confirmed,” or “no”), passive smoking during pregnancy (“yes” or “no”), drinking alcohol during pregnancy (“yes” or “no”), history of disease (“yes” or “no”), history of mental illness (“yes” or “no”), and depressive symptoms that continue for more than two weeks such as “inability to sleep,” “feeling irritated/frustrated,” “being emotional,” and “not feeling like doing anything” in the previous year (“yes” or “no”). Marital status was defined as “married” when pregnant women were legally married and “unmarried” when they were not. Pregnancy progress was defined as “good” when mothers reported their pregnancies were going well. Returning to parents’ home for delivery, called “*satogaeri bunben/shussan*,” is traditionally common in Japan and indicates that mothers have support from their parents after delivery and fathers do not stay with mothers and their child for some period of time (i.e., 1 to 2 months). History of diseases was defined “yes” if pregnant women had experienced at least one of the following diseases: heart disease, high blood pressure, chronic nephritis, diabetes, hepatitis, and mental illness. 

For analysis, maternal age was categorized as “younger than 20 years old”, “20–24 years old”, “25–39 years old”, or “40 years and older,” based on previous studies showing that mothers aged 24 years or younger and those aged 40 years or older have an increased risk for infant abuse [20,21]. Late registration was defined as registration of pregnancy notification forms at 12 weeks or later. Mothers’ feelings when notified of the pregnancy were classified into three categories: “happy”, “unexpected but happy”, “unexpected and puzzled/did not know what to do/no feeling/other.”

When a response to a question was missing, this was indicated as “not answered” and included in the analysis. In this study, an indication of “not answered” could mean different things depending on the item and municipality. For example, not answering a particular question can be considered a risk, but it could also have been related to the data digitization process, which varied among municipalities. Therefore, the “not answered” category was combined with the reference category when the number of participants who did not provide an answer was less than five. Additionally, some of the questions (i.e., marital status, pregnancy progress, history of infertility treatment, returning to parents’ home for delivery, having worries or anxiety, passive smoking during pregnancy, history of disease) were missing in some municipalities because not all questions and responses were electronically recorded in all municipalities, even though the same questions were included in pregnancy notification forms across Aichi Prefecture. Responses to these questions were indicated as “not investigated.”

### 2.3. Ethics

This study was approved by the Ethics Committee at the National Center for Child Health and Development (Study ID: 716) in Tokyo, Japan. Because we used administrative data, written informed consent was not obtained, which was approved by the Ethics Committee.

### 2.4. Statistical Analysis

First, crude exact logistic regression, which can be used to analyze small or unbalanced binary data [22], was conducted to examine whether prenatal characteristics differed between children who were registered with RCCRC and children who were not (Table 1). Based on these regressions, statistically significant variables were selected and examined for associations with child maltreatment, with adjustment for the other variables. 

Multiple exact logistic regression was performed using selected risk factors. Using odds ratios (ORs) obtained from this multiple exact logistic regression as weights, a risk score was calculated [23]. A weight of 3 was applied when the OR was 10 or larger, a weight of 2 was applied when the OR was between 5 and 10, and no weight was applied to a score when the OR was smaller than 5. A cut-off score that reliably predicts risk for child maltreatment by age three was determined. Sensitivity analysis was also conducted to calculate a risk score using all risk factors. All analyses were conducted using Stata/SE v14.2 software (StataCorp, College Station, TX, USA).

## 3. Results

### 3.1. Characteristics of the Participants in This Study

Table 1 presents the characteristics of the sample. A total of 11 children (1.2%) were registered with RCCRC either as children in need of protection (*yohogojido*) or those requiring social support (*yoshienjido*) by the age of three. Nearly half the children were born to married mothers and 12% were born to mothers under the age of 25. Firstborn children accounted for 44.6%. Regarding prenatal circumstances, 6.2% of mothers registered their pregnancy at 12 gestational weeks or later and nearly 30% had unintended pregnancy. Meanwhile, 6.6% had experienced artificial abortion once or more, 35.5% indicated that they planned to return to their parents’ home for delivery, while 1.5% indicated that they did not have anyone to help when in need. Approximately 30% had worries or anxiety at the time of pregnancy registration, and 7.4% had depressive symptoms in the previous year. Further, 2.6% of mothers smoked tobacco and 0.9% drank alcohol during pregnancy, while 9.6% reported passive smoking. More than 10% had a history of some kind of disease, and nearly 3% had a history of mental illness.

### 3.2. Association between Parental Risk Factors and Child Maltreatment

Results of crude exact logistic regression analysis of each characteristic are also shown in Table 1. Prenatal risk factors that were significantly associated with registration with RCCRC included unmarried status (OR 16.54; 95% CI, 1.15–236.93), history of artificial abortion (OR 8.54; 95% CI, 1.78–34.83), and smoking during pregnancy (OR 12.10; 95% CI, 1.13–73.05).

Table 2 shows the results of multiple exact logistic regression analyses of the following selected risk factors, conducted based on the results of the previous crude logistic regression analysis: marital status, history of artificial abortion, and smoking during pregnancy. After adjusting for each factor, children of mothers with a history of artificial abortion were 5.82 times more likely to be registered with RCCRC than their counterparts (95% CI, 1.08–26.05). In contrast, maternal marital status (unmarried: OR 12.14; 95% CI, 0.72–196.16; not answered: OR 8.76; 95% CI, 0.14–175.81; not investigated: OR 3.10; 95% CI, 0.54–31.74) and smoking during pregnancy (stopped after pregnancy was confirmed: OR 3.35; 95% CI, 0.53–16.23; yes: OR 5.30; 95% CI, 0.37–42.71) had no significant association with RCCRC registration in multiple logistic regression analysis.

### 3.3. Prenatal Risk Scores

Prenatal risk scores were calculated based on the results of multiple logistic regression as follows:

Prenatal risk score = 3*unmarried status + 2*marital status not answered + 1*marital status not investigated + 2*history of artificial abortion + 1*stopped smoking after pregnancy was confirmed + 2*smoking during pregnancy.

Using this formula, 346 mothers (38.7%) scored zero, 391 (43.8%) scored one, 81 (9.1%) scored two, 54 (6.0%) scored three, 14 (1.6%) scored four, 6 (0.7%) scored five, and one mother scored seven. Sensitivity, specificity, the overall rate of correct classification, and positive and negative predictive values were calculated and compared, as shown in Table 3. The area under the receiver operating characteristic curve (AUC) of the prenatal risk score was 0.805 (95% CI, 0.660–0.950), which suggests high predictive power. These results suggest that the cut-off prenatal risk score should be 2 (sensitivity = 72.7%, specificity = 83.2%, overall rate of correct classification = 83.1%).

The risk score calculated from all predictors as sensitivity analysis also ranged from 0 to 9, and the AUC of the score was 0.795 (95% CI, 0.622–0.967). It suggested the cut-off score as 4 (sensitivity = 63.6%, specificity = 81.6%, overall rate of correct classification = 81.4%). The risk score calculation model based on selected predictors showed higher predictive power.

## 4. Discussion

To our knowledge, this is the first population-based prospective cohort study in Japan to investigate prenatal risk factors for child maltreatment during the first three years of life, using administrative data from a pregnancy registration system and registration with RCCRC. We found that unmarried status, history of artificial abortion, and smoking during pregnancy significantly predicted child maltreatment. Additionally, prenatal risk scores calculated from these factors indicated high predictive power at a cut-off score of two.

Risk factors identified in this study are largely consistent with those of previous population-based studies on prenatal risk factors for child maltreatment during the first few years of life, including unmarried status, smoking during pregnancy, and history of artificial abortion [11,12,13,14,15]. Unmarried status can lead to social instability, which was previously identified as a risk factor for family violence [15,24]. In Japan, where the majority of children are born to married couples, having nonmarital births can be particularly challenging [25,26]. Moreover, previous research suggests that smoking during pregnancy and history of artificial abortion can be considered markers or proxies for other maternal situations or characteristics that impose a risk for child maltreatment [11,13]. For example, maternal smoking during pregnancy has been shown to be associated with lower levels of education and socioeconomic status [27,28]. Similarly, a history of artificial abortion, especially when repeated, may imply maternal experience with IPV [29,30,31]. Because IPV was not directly assessed in the pregnancy notification, the percentage of the artificial abortions which are related to IPV is unknown in this study. However, a previous study in Italy revealed that 22.3% of women with repeated induced abortion and 14.8% of women experiencing their first induced abortion reported past or current IPV [31]. Although only 1.5% in our sample repeated artificial abortions, IPV could be the reason behind the abortions. Although active smoking during pregnancy and history of artificial abortion are likely to be underreported [11,32], our findings suggest that it is important to assess these factors and provide support to pregnant women who report these risk factors through maternal and child health services.

Some previously identified risk factors were not significantly associated with child maltreatment in this study. For example, while young maternal age, often younger than 20 years old, has been reported to be associated with child maltreatment [11,15], this association was not observed in this study. The discrepancy may be attributed to the small number of young mothers, such as the 1.5% of teen mothers, in this sample due to the increase in late childbearing in Japan. Further, a study using a large UK cohort indicated that children of mothers with unintended pregnancy were 1.5 times more likely to be registered with child protection services for maltreatment [33]. However, in our study, unintended pregnancy, including those in women who reported being happy and unhappy with the unexpected pregnancy, did not predict future maltreatment. This discrepancy may be due to social desirability bias and cultural differences, as pregnancy notification forms are often completed and submitted in person. There is also a possibility that some unintended pregnancies were terminated by artificial abortion. In Japan, artificial abortion is legal under certain conditions, such as when it is difficult to continue pregnancy due to women’s health and economic problems and when they are sexually abused. These conditions are subjectively self-reported by pregnant women. Additionally, contraception is heavily dependent on males, given that the prevalence of oral contraceptive pill use is low and a major contraceptive method is condoms. Taken together, these situations lead to a high rate of artificial abortion in Japan (7.0 per 1000 females aged 15 to 49 in 2013) [34], which may contribute to a null association between unintended pregnancy and child maltreatment. Similarly, the association between having two or more children and child maltreatment was not statically significant in the current study. This is in contrast to previous studies, which demonstrated that a subsequent child was 1.5 times more likely to be maltreated [11,14]. That Japan has a lower proportion of families with two or more children than countries in which similar studies have been conducted (i.e., United States) may explain this result. Alcohol use during pregnancy is another risk factor often identified in previous research [14,15] that was not found to be significantly associated with child maltreatment in this study. In this study, a smaller number of pregnant women reported drinking than smoking during pregnancy, which may explain the statistically insignificant association for the former factor. There was also a lower prevalence of alcohol use during pregnancy (0.9%) in this study than previous studies in Japan (4.6% to 9.1%) [35,36]. Because this study only included participants from five non-urban municipalities in Aichi Prefecture, further research using a nationally representative sample is warranted to elucidate the prevalence of alcohol use during pregnancy and its association with future maltreatment.

Some prenatal risk factors identified in previous research were not measured in this study due to differences in cultural context or pregnancy registration systems. For example, substance use, IPV, maternal education, and financial situation (i.e., household income and public aid) are culturally difficult to inquire about in universal assessments conducted by municipalities. Further, prenatal care is not queried in pregnancy notification forms because mothers are recommended to register pregnancy notification forms as early as possible once pregnancy is confirmed in order to receive prenatal care. However, it should be noted that not all pregnant women registering their pregnancy at municipalities necessarily attend all their prenatal health checks.

In addition to identifying prenatal risk factors for child maltreatment, it is important to identify the number of risk factors that indicate the need for support. A person with multiple risk factors is known to have increased risk for child maltreatment, with research showing a dose–response relationship between the number of risk factors and child maltreatment [11,13,14]. With the aim of risk screening, previous population-based studies categorized participants into risk groups and investigated the proportion of child maltreatment cases in each group. A study using the Pregnancy Risk Assessment Monitoring System in Alaska found that those with two or more risk factors, which was 32% of the population, accounted for 75% of all maltreatment cases [14]. Similarly, another study using the Florida Healthy Start prenatal risk screening instrument found that those with three or more risk factors, which was 13% of the population, accounted for 50.3% of infant maltreatment cases [13]. However, these previous studies did not account for the effect size of each risk factor using weights. Calculating risk scores that were weighted based on odds ratios obtained from logistic regression analysis, our study revealed that pregnant women who had a risk score of two or higher, which was 17.5% of the sample, accounted for 72.7% of all RCCRC registration cases.

Our prenatal risk score can be easily utilized in Japanese maternal and child health settings to support pregnant women and prevent child maltreatment because we employed assessment items used in pregnancy notification forms currently being adopted by many municipalities. In the absence of evaluation for future maltreatment, these assessment items have been used to identify at-risk pregnant women and to provide them with support through maternal and child health services, including telephone calls, interviews, and home visitation by public health nurses. Our findings provide the first evidence that prenatal risk factors measured in Japanese pregnancy notification forms can predict actual child maltreatment during the first three years of life. This may help to promote the use of evidence-based prenatal risk assessment and begin prevention efforts from an early stage of pregnancy based on a universal prenatal risk assessment. This risk score may be useful for other countries which employ a pregnancy registration system at an administration office; further validation of the predictability of the risk score for child maltreatment is needed.

There are several limitations of this study. First, child maltreatment in this study was measured based on registration with RCCRC, which may only reveal severe child maltreatment cases. It should be noted that not all maltreatment cases are detected and registered with RCCRC, with research revealing that official records underestimate the prevalence of child maltreatment [4,37,38]. Moreover, some groups, such as unmarried and young mothers, may be overrepresented among RCCRC registration cases due to social class bias or social disadvantages [39,40], especially with a lack of fixed standards for RCCRC registration [41]. On the other hand, these mothers might actually have a higher risk for child maltreatment, as some previous studies suggested [39,42]. It is important to examine the association between risk factors and child maltreatment using objective measures of child maltreatment, because RCCRC registration is one of the limited sources of objective records in Japan, and our findings will be useful for predicting child maltreatment cases registered with RCCRC. Second, administrative data on RCCRC registration were only available for the first three years in this study. We believe that predicting and preventing maltreatment for the first three years is particularly important, given that children under 3 years old accounted for almost 70% of the deaths from child maltreatment (except filicide–suicide) in 2007–2016 in Japan [10]. However, future research is warranted to examine the longer-term effects of prenatal factors on child maltreatment. Third, although it is reported that more than 99% of women register their pregnancy at municipalities [18], not all pregnant women do so. High-risk pregnant women, such as those who undergo delivery with no prenatal care and those who move from or to another municipality during pregnancy or after birth, were less likely to be included in our sample, which may have led to an underestimation of the association between prenatal risk factors and child maltreatment. Fourth, there may have been potential unadjusted confounders that were not measured in pregnancy notification forms. Pregnant women’s educational attainment, receipt of public assistance, existence of IPV, and inadequate prenatal care have been shown to be associated with child maltreatment [11,12,13,14,15,43] and other prenatal risk factors such as smoking and drinking alcohol during pregnancy [44,45,46,47]. Although significant risk factors identified in this study may merely be proxy variables for low socioeconomic status and do not have causal relationships with child maltreatment [13], they can be useful for identifying pregnant women in need of support. Fifth, data on some risk factors were not available in some municipalities due to limited digitization of data. This caused the high percentage of missing data on marital status, which was one of the main predictors of the risk for child maltreatment. We categorized these missing data as “not investigated” and distinguished them from “not answered” data. As the percentages of “not investigated” data were not very different between RCCRC registered cases (54.5%) and non-registered cases (46.6%) and it is expected that the percentages of unmarried women do not vary from municipality to municipality, we assume that the high percentage of “not investigated” data may not critically affect our findings on marital status. However, further research using complete data on all risk factors is needed. Finally, our findings are not generalizable because we used data from only five municipalities in Aichi Prefecture, Japan, where data from pregnancy notification forms were able to link with administrative records of registration with RCCRC. Aichi Prefecture includes Nagoya-city, an ordinance-designated city, and has the fourth largest population [16] and third highest prefectural income among Japanese prefectures in 2013 [17]. The five municipalities are suburb areas in Aichi Prefecture. Due to the lack of national average data for pregnancy registration forms, it was not possible to compare the distributions of prenatal risk factors observed in this study with the national norms. Future studies should explore prenatal risk factors for child maltreatment using a nationally representative sample of Japanese pregnant women when data linkage between pregnancy notification forms and registration with RCCRC is available in other municipalities.

## 5. Conclusions

In conclusion, this population-based prospective feasibility study demonstrated that prenatal risk factors assessed in pregnancy notification forms can predict child maltreatment by the age of three, using objective measures of child maltreatment. This suggests that risk assessment at universal pregnancy registration in Japan may be effective for identifying at-risk pregnant women and providing support at an early stage of pregnancy, which may lead to better outcomes for pregnant women, their children, and their families.

## Figures and Tables

**Table 1:** Characteristics of sample (*n* = 893).

	ALL		No Reported Child Maltreatment		Reported Child Maltreatment		Crude Odds Ratio ^a^	
	(*n* = 893)		(*n* = 882)		(*n* = 11)			
	*n*	%	*n*	%	*n*	%	OR	95%CI
**Marital status**								
• Married	423	47.4	421	47.7	2	18.2	Reference	
• Unmarried	27	3.0	25	2.8	2	18.2	**16.54**	**1.15−236.93**
• Not answered	26	2.9	25	2.8	1	9.1	8.32	0.14−165.00
• Not investigated	417	46.7	411	46.6	6	54.5	3.07	0.54−31.26
**Maternal age**								
• <20	13	1.5	12	1.4	1	9.1	10.27	0.21−95.77
• 20–24	94	10.5	91	10.3	3	27.3	4.09	0.65−19.56
• 25–39	753	84.3	747	84.7	6	54.5	Reference	
• ≥40	33	3.7	32	3.6	1	9.1	3.88	0.08−33.46
**Late Registration (≥ 12w)**								
• No	812	90.9	803	91.0	9	81.8	Reference	
• Yes	55	6.2	53	6.0	2	18.2	3.36	0.34−16.82
• Not answered ^b^	26	2.9	26	2.9	0	0	2.51	0−16.55
**Pregnancy Progress**								
• Good/Not answered ^c^	557	62.4	549	62.2	8	72.7	Reference	
• Not good ^b^	10	1.1	10	1.1	0	0	5.13	0−37.06
• Not investigated	326	36.5	323	36.6	3	27.3	0.64	0.11−2.68
**Birth Order**								
• Subsequent child/Not answered ^c^	495	55.4	488	55.3	7	63.6	Reference	
• First child	398	44.6	394	44.7	4	36.4	0.71	0.15−2.80
**History of Artificial Abortion**								
• No/Not answered ^c^	834	93.4	827	93.8	7	63.6	Reference	
• Yes	59	6.6	55	6.2	4	36.4	**8.54**	**1.78−34.83**
**Infertility treatment**								
• No	696	77.9	686	77.8	10	90.9	Reference	
• Yes ^b^	67	7.5	67	7.6	0	0	0.74	0−4.68
• Not answered ^b^	6	0.7	6	0.7	0	0	8.65	0−66.08
• Not investigated	124	13.9	123	13.9	1	9.1	0.56	0.01−3.99
**Feeling at Pregnancy**								
• Happy	647	72.5	640	72.6	7	63.6	Reference	
• Unexpected, but happy	185	20.7	183	20.7	2	18.2	1.00	0.10−5.31
• Unexpected and puzzled/do not know what to do/no feeling/other	54	6.0	53	6.0	1	9.1	1.72	0.04−13.83
• Not answered	7	0.8	6	0.7	1	9.1	14.98	0.29−153.76
**Returning to Parents’ Home for Delivery**								
• Yes	317	35.5	315	35.7	2	18.2	Reference	
• No	440	49.3	432	49.0	8	72.7	2.91	0.58−28.34
• Not answered ^b^	12	1.3	12	1.4	0	0	11.0	0−145.61
• Not investigated	124	13.9	123	13.9	1	9.1	1.28	0.02−24.79
**Having Someone Who Can Help in Need**								
• Yes	870	97.4	861	97.6	9	81.8	Reference	
• No	13	1.5	12	1.4	1	9.1	7.92	0.17−65.92
• Not answered	10	1.1	9	1.0	1	9.1	10.53	0.22−92.22
**Having Worries/Anxiety**								
• No	418	46.8	414	46.9	4	36.4	Reference	
• Yes	264	29.6	259	29.4	5	45.5	2.00	0.43−10.15
• Not answered ^b^	9	1.0	9	1.0	0	0	8.97	0−78.00
• Not investigated	202	22.6	200	22.7	2	18.2	1.03	0.09−7.29
**Smoking Before/During Pregnancy**								
• No/Not answered ^c^	776	86.9	770	87.3	6	54.5	Reference	
• Stopped after pregnancy was confirmed	94	10.5	91	10.3	3	27.3	4.22	0.67−20.16
• Yes	23	2.6	21	2.4	2	18.2	**12.10**	**1.13−73.05**
**Passive Smoking During Pregnancy**								
• No/Not answered ^c^	481	53.9	476	54	5	45.5	Reference	
• Yes	86	9.6	83	9.4	3	27.3	3.43	0.52−18.01
• Not investigated	326	36.5	323	36.6	3	27.3	0.88	0.14−4.58
**Drinking Alcohol During Pregnancy**								
• No	874	97.9	864	98.0	10	90.9	Reference	
• Yes	8	0.9	7	0.8	1	9.1	12.21	0.25−111.41
• Not answered ^b^	11	1.2	11	1.2	0	0	5.8	0−40.14
**Past Disease History**								
• No/Not answered ^c^	476	53.3	470	53.3	6	54.5	Reference	
• Yes	91	10.2	89	10.1	2	18.2	1.76	0.17−10.0
• Not investigated	326	36.5	323	36.6	3	27.3	0.73	0.12−3.44
**Past Disease History: Mental Illness**								
• No	310	34.7	307	34.8	3	27.3	Reference	
• Yes	25	2.8	24	2.7	1	9.1	4.23	0.08−55.05
• Not answered	434	48.6	428	48.5	6	54.5	1.43	0.30−8.93
• Not investigated	124	13.9	123	13.9	1	9.1	0.83	0.02−10.48
**Depressive Symptoms in the Previous Year**								
• No	820	91.8	811	92.0	9	81.8	Reference	
• Yes	66	7.4	64	7.3	2	18.2	2.81	0.29−14.00
• Not answered ^b^	7	0.8	7	0.8	0	0	9.70	0−72.61

^a^ Exact logistic regression was performed. ^b^ Median unbiased estimates were computed because no one in this category was registered with Regional Councils for Children Requiring Care. ^c^ When the number of participants who did not provide an answer was less than five, the “not answered” category was combined with the reference category. **Bold**: *p* < 0.05; OR, odds ratio; CI, confidence interval.

**Table 2:** Odds ratios for registration with Regional Councils for Children Requiring Care.

	Multiple	
	OR	95% CI
**Marital Status**		
• Married	Reference	
• Unmarried	12.14	0.72−196.16
• Not answered	8.76	0.14−175.81
• Not investigated	3.10	0.54−31.74
**History of Artificial Abortion**		
• No/Not answered	Reference	
• Yes	**5.82**	**1.08−26.05**
**Smoking Before/During Pregnancy**		
• No/Not answered	Reference	
• Stopped after pregnancy was confirmed	3.35	0.53−16.23
• Yes	5.30	0.37−42.71

Bold: *p* < 0.05; OR, odds ratio; CI, confidence interval.

**Table 3:** Prediction parameters for risk scores.

Score	Sensitivity	Specificity	Overall Rate of Correct Classification	Positive Predictive Value	Negative Predictive Value
0	100.0	0.0	1.2		
1	90.9	39.1	39.8	1.8	99.7
2	72.7	83.2	83.1	5.1	99.6
3	36.4	92.0	91.3	5.3	99.1
4	27.3	98.0	97.1	14.3	99.1
5	18.2	99.4	98.4	28.6	99.0
7	9.1	100.0	98.9	100.0	98.9
>7	0.0	100.0	98.8		

## Data Availability

Deidentified individual participant data will not be made available.
